# Pneumatization pattern of the temporal bone with volumetric assessment and its impact on the frequency of cerebrospinal fluid leak in patients undergoing vestibular schwannoma surgery via the retrosigmoid approach

**DOI:** 10.3389/fneur.2024.1504999

**Published:** 2024-11-12

**Authors:** Karol Zaczkowski, Rafał Wójcik, Krzysztof Tybor, Dariusz Jan Jaskólski, Karol Wiśniewski

**Affiliations:** Department of Neurosurgery and Neurooncology, Medical University of Lodz, Barlicki University Hospital, Lodz, Poland

**Keywords:** vestibular schwannoma, retrosigmoid approach, temporal bone, pneumatization, computed tomography, acoustic neuroma

## Abstract

**Introduction:**

Vestibular schwannomas are benign tumors that account for 8% of all intracranial tumors. Due to their location in the cerebellopontine angle and internal auditory canal, access to these lesions requires disruption of the temporal bone. The pneumatization of this bone varies between individuals, and literature reports suggest that it may influence the frequency of cerebrospinal fluid leaks. In our study, we assessed whether the pneumatization of the temporal bone differs in individuals with or without cerebrospinal fluid leak.

**Methods:**

We conducted a retrospective analysis that initially included 143 individuals, of whom 103 were ultimately qualified for the study. We analyzed the clinical data of the patients and the radiological characteristics of the pneumatization of the temporal bone using volumetric methods, additionally relying on classifications of temporal bone pneumatization.

**Results:**

Pneumatization of the temporal bone in patients operated on for vestibular schwannoma with cerebrospinal fluid leakage was significantly higher compared to the group without leakage (Right: 11.15 [IQR 8.93–12.83] vs. 13.25 [IQR 10.15–15.53]; *p* = 0.040; Left: 10.95 [IQR 9.5–12.26] vs. 14.4 [IQR 13.03–15.7]; *p* = 0.012). Additionally, a higher degree of pneumatization of the petrous apex was significantly more frequent in the group with cerebrospinal fluid leak in both left (*p* < 0.001) and right (*p* < 0.001) side.

**Discussion:**

The analyzed data suggest that greater pneumatization of the temporal bone and a higher degree of petrous apex pneumatization in the classification of temporal bone pneumatization may be associated with an increased risk of cerebrospinal fluid leak. To draw causal inferences, prospective studies in this area are necessary.

## Introduction

Vestibular schwannomas (*VS*) are benign tumors originating from Schwann cells, accounting for 7–9% of intracranial tumors they are the most common tumors in the cerebellopontine angle (1). Their incidence ranges between 3 and 5 cases per 100,000 people per year, and the median age of onset is 56–60 years (2, 3). The treatment strategy for *VS* varies, taking into consideration not only the benign nature of these tumors, but also the fact that their location and potential for further growth pose a significant danger to the patient. Current management options include active surveillance, microsurgical treatment, or radiotherapy. The most critical factor in choosing a treatment method is the size of the tumor. For large tumors causing brainstem compression, hydrocephalus, trigeminal neuralgia, or other neuropathies, surgery is the treatment of choice (4). One of the possible approaches to *VS* is the retrosigmoid approach. Its selection may be influenced by not only tumor size but also the patient’s hearing status, as it allows for the preservation of the inner ear structures and consequently hearing, unlike other techniques such as the translabyrinthine approach. The surgical risk is directly proportional to the size of the tumor. The most common complications include postoperative hearing loss, temporary or permanent facial nerve dysfunction, wound healing issues caused by cerebrospinal fluid (CSF) leakage, and aseptic meningitis. CSF leak after *VS* surgery can present in two forms: one is CSF rhinorrhea, where fluid leaks from the nose, and the other involves fluid accumulation under the wound, leading to poor wound healing and CSF discharge through the wound. CSF leak most commonly occur between the 2nd and 7th day after surgery (5). They are usually managed with external lumbar drainage, but in some cases, reoperation is necessary. Several studies in the literature point to factors predisposing to CSF leak, such as BMI, age, craniectomy, or reoperation (6–8). Shew et al. published a study in 2018, noting that pneumatization of the petrous apex (PA) may be associated with more frequent CSF leak through the nose and wound. Currently, there is a classification of temporal bone pneumatization types based on computed tomography, dividing the PA pneumatization into four groups, mastoid pneumatization into three groups, and infralabyrinthine pneumatization into three groups (9). The impact of these subtypes on CSF leak rates has never been evaluated, and the volumes of temporal bone pneumatization in these subtypes are unknown. As a result, the classification remains subjective; while it may suggest a more pneumatized bone type, without volumetric measurements of the pneumatized cells, there is no certainty.

The main objective of our study was to investigate whether the degree of temporal bone pneumatization affects the frequency of CSF leak from the nose or the wound. We hypothesize that an increase in the pneumatization of the temporal bone may be an independent risk factor for CSF leak. In order to validate our theory, we conducted a retrospective analysis of a group of patients who underwent vestibular schwannoma surgery, aiming to validate the temporal bone pneumatization classification by supplementing it with volumetric measurements for each pneumatization subtype.

## Materials and methods

### Clinical data

**Figure 1:**
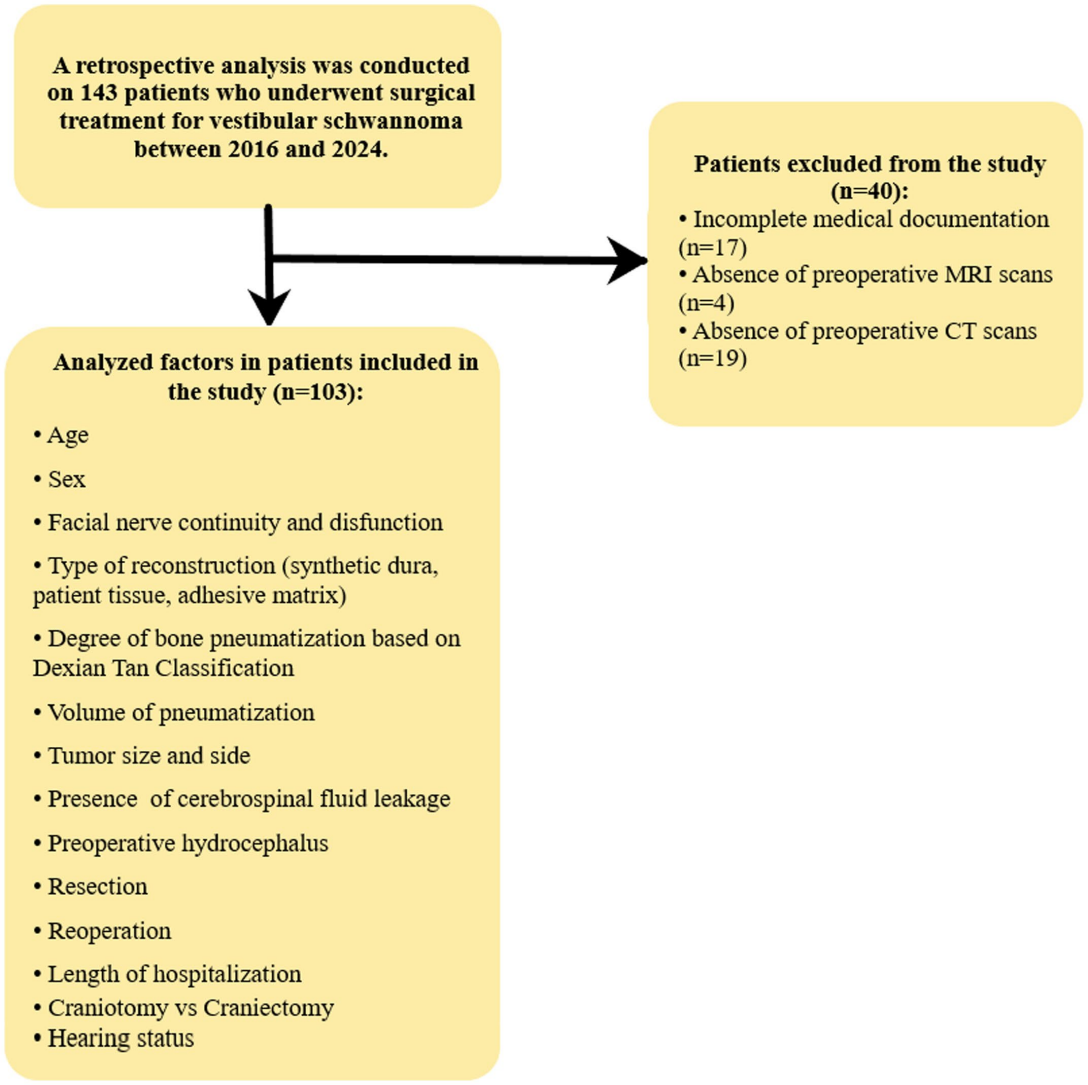
illustrates the decision-making process for qualifying patients for the study.

We analyzed the medical records of 143 patients who underwent surgery between 2016 and 2024 at the Department of Neurosurgery and Neurooncology at Norbert Barlicki Memorial Teaching Hospital No. 1 of whom 103 were included in the study. Patients with incomplete medical records or missing MRI and CT imaging studies were excluded from the analysis. The analyzed factors included age, sex, facial nerve continuity and disfunction, type of reconstruction (synthetic dura, patient tissue, adhesive matrix), degree of bone pneumatization based on Dexian Tan classification, volume of pneumatization, tumor size and side, presence of cerebrospinal fluid leakage, extent of resection, whether the patient underwent reoperation, the length of hospitalization and patient’s hearing status was evaluated by an otolaryngologist based on additional tests and physical examination. Based on Gardener and Robertson modified hearing classification, hearing impairments were generally divided into three subgroups: “good excellent,” “serviceable” and “non- serviceable.” For “good excellent,” the pure tone audiogram (PTA) is 0–30 dB, and the speech discrimination is 70–100%. For “serviceable,” the PTA ranges from 31 to 50 dB, with speech discrimination between 50 and 69%. For “non-serviceable,” the PTA is 51 dB-nontestable, and the speech discrimination ranges from 49 to 0%. A detailed description of the analyzed data and the patient inclusion criteria is illustrated in Figure 1.

### Radiological analysis

**Figure 2:**
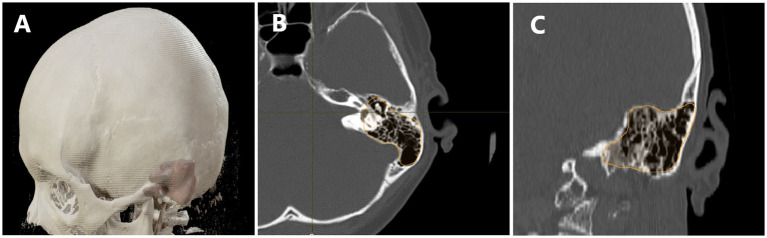
Demonstrates the use of volumetric analysis in assessing cranial bone pneumatization. In panel **(A)** we see a visualization of the model with the pneumatization of the temporal bone overlaid. **(B)** Shows a transverse section with the pneumatized area outlined, while panel **(C)** presents a coronal section with the pneumatized area highlighted.

The radiological analysis was conducted using BrainLab software, based on CT and MRI scans. Volumetric assessment of tumor size was performed using preoperative MRI, while the volumetric analysis of temporal bone pneumatization, along with an evaluation of the pneumatization pattern, was conducted based on preoperative CT scans. The radiological analysis was performed by two independent researchers, and the results represent the average of their measurements. A total of 103 preoperative MRI scans and 103 CT scans were analyzed. Figure 2 illustrates the use of BrainLab software’s volumetric analysis in performing the measurements.

### Classification adaptation

**Figure 3:**
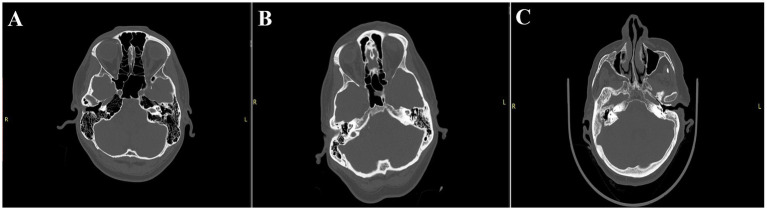
Illustrates selected subtypes evaluated in the Dexian Tan classification. **(A)** Indicates a significant type of pneumatization: 4th degree apex pneumatization, 4th degree mastoid pneumatization, and 1st degree infralabyrinthine pneumatization, with a total score of 11, classified as significant pneumatization. **(B)** Presents the most typical pneumatization: 2nd degree apex pneumatization, 4th degree mastoid pneumatization, and 2nd degree infralabyrinthine pneumatization, with a total score of 7, classified as moderate pneumatization. **(C)** Shows weak pneumatization: 1st degree apex pneumatization, 1st degree mastoid pneumatization, and 2nd degree infralabyrinthine pneumatization, with a total score of 4, classified as poor pneumatization.

The Dexian Tan et al. classification system is based on the evaluation of three regions of the temporal bone. The analyzed areas include pneumatization of the petrous apex, mastoid pneumatization, and infralabyrinthine pneumatization. For the first two areas, a total of four grades are distinguished, where grade I represents hypopneumatization and grade IV represents hyperpneumatization. In the infralabyrinthine pneumatization subgroup, there are three subtypes, with grade I indicating good pneumatization and grade III indicating the absence of air cells. To facilitate the statistical analysis, we decided to create a scoring system based on this classification. In this system, the maximum score in the first two areas is awarded for grade IV, while in the infralabyrinthine region, the highest score is given for grade I. This approach provides a clearer picture of the overall pneumatization of the temporal bone. Additionally, we performed analyses for each region individually to determine whether any specific area is particularly associated with the frequency of CSF leaks (Figure 3).

In order to compare the mean volume of pneumatization between groups based on classification, an assessment of normality distribution was conducted in the subgroups. For the left side, the distribution was normal across all subgroups; therefore, a homogeneity of variance test was performed, which confirmed the homogeneity of variances. Subsequently, a one-way ANOVA was conducted along with the Post-Hoc Scheffé test. On the right side, the distribution of pneumatization volume was different from normal; thus, the Kruskal-Wallis test was applied, followed by pairwise comparison analysis with Bonferroni correction.

### Ethical approval

The study protocol was created according to the Declaration of Helsinki and Good Clinical Practice (GCP). It was further approved by the local Ethical Committee (number RNN/279/23/KE).

### Statistical analysis

Statistical analysis was conducted on both quantitative and qualitative variables. For the quantitative variables, normality was assessed using the Shapiro–Wilk test. If the data followed a normal distribution, Student’s t-test was used for comparison of the quantitative variables. For data that did not follow a normal distribution, the Mann–Whitney U test was applied. Nominal variables were evaluated using the Chi-squared test, and when the assumptions for this test were not met, Fisher’s exact test was used. Quantitative data that conformed to a normal distribution were presented as mean and standard deviation, while variables with a non-normal distribution were presented as median and interquartile range. For nominal variables, percentages were reported for the study group. The statistical analysis was conducted using IBM SPSS Statistics v 27.0.

## Results

### Clinical and radiological data

A total of 103 patients were included in the study, and for the purpose of analysis, they were divided into a control group and a CSF leak group. The clinical data and statistical comparison are summarized in Table 1. Statistical analysis showed that the occurrence of CSF leakage was more frequently associated with preoperative hydrocephalus (8 cases in the control group vs. 15 cases in the CSF leak group; *p* < 0.001), as well as pneumatization of the temporal bone on the left side (10.65 [IQR 9.4–12.1] vs. 13.65 [IQR 11.32–15.27]; *p* = 0.002) and on the right side (10.6 [IQR 8.71–12.45] vs. 14.4 [IQR 12.4–17.3]; *p* < 0.001). Interestingly, when considering the Dexian Tan classification, the frequency of subgroups in the three pneumatization regions was compared, the petrous apex pneumatization appeared to be most strongly associated with CSF leak, with significant differences in both the left (*p* < 0.001) and right sides (*p* < 0.001) between the CSF leak and control groups. In the CSF leak group, there was a higher frequency of subtypes III (L: 9; 36% vs. 6; 7.7%; R: 9; 36% vs. 6; 7.7%) and IV (L: 5; 16% vs. 4; 6.4%; R: 3; 16% vs. 4; 3.8%). Mastoid pneumatization differed between the CSF leak and control groups only on the left side (*p* = 0.023), while infralabyrinthine pneumatization did not show significant differences on either side (L: *p* = 0.083; R: *p* = 0.168). This analysis includes both sides, regardless of which side the tumor was located on. The collected data are presented in summary form in Table 1.

**Table 1:** The clinical and radiological data.

	Control group (*n* = 78)			CSF Leak group (*n* = 25)			
Age	54.9 (±14.4)			51 (±15.9)			*p* = 0.313*
Sex (M – male; F – female; *n*%)	M – 29 (37.18%)	F – 49 (62.8%)		M – 8 (32%)		F – 17 (68%)	*p* = 0.811**
Anatomical continuity of the facial nerve (A – Preserved B – Not preserved; *n*%)	A – 71 (91%)	B – 7 (9%)		A – 22 (88%)		B – 3 (12%)	*p* = 0.657**
Facial nerve (VII) dysfunction according to the House-Brackmann scale. (*n*%)	Pre-operative	Post-operative		Pre-operative	Post-operative		Post-operative: *p* = 0.956**
	I – 76 (97.4%) II – 1 (1.3%) III – 1 (1.3%) IV – 0 V – 0 VI – 0	I – 22 (28.2%) II – 3 (3.8%) III – 11 (14.1%) IV – 29 (37.2%) V –2 (2.6%) VI – 11 (14.1%)		I – 19 (96%) II – 1 (4%) III – 0 IV – 0 V – 0 VI – 0	I – 8 (32%) II – 1 (4%) III – 3 (12%) IV – 8 (32%) V – 0 VI – 5 (20%)		
Type of reconstruction (A - Synthetic dura B - Adhesive matrix C - Patient tissues; *n*%)	A – 7 (8.9%)	B – 35 (44.9%)	C – 36 (46.2%)	A – 4 (16%)	B – 13 (52%)	C – 8 (32%)	*p* = 0.387**
Pre-operative hydrocephalus (*n*%)	8 (10.3%)			15 (60%)			*p* < 0.001**
30 day - Mortality (*n*%)	2 (2.6%)			1 (4%)			
Length of hospitalization (days; median with IQR)	9 (IQR 8–11.75)			12 (IQR 9–18.5)			*p* = 0.007*
Resection (A – Total resection B – Subtotal resection; *n*%)	A – 61 (78.2%)	B – 17 (21.8%)		A – 17 (68%)		B – 8 (32%)	*p* = 0.300**
A – Craniotomy B – Craniectomy (*n*%)	A – 52 (66.7%)	B – 26 (33.3%)		A – 13 (52%)		B – 12 (48%)	*p* = 0.186**
Hearing loss (A – Non-serviceable I B – serviceable C – good-excellent; *n*%)	Pre-operative			Pre-operative			*p* = 0.741**
	A – 33 (42.3%)	B – 41 (52.6%)	C - 4 (5.1%)	A – 13 (52%)	B – 12 (48%)	C – 0	
	Post-operative			Post-operative			p = 0,793**
	A – 56 (75.6%)	B – 19 (24.4%)	C – 0	A – 18 (72%)	B – 7 (28%)	C – 0	
Reoperation (*n*%)	7 (9%)			14 (56%)			*p* < 0.001**
Tumor volume (cm3; median with IQR)	10.45 (IQR 5.44 – 16.2)			10.85 (IQR 8.83 – 15.52)			*p* = 0.309*
Side of tumor (*n*%)	R – 38 (48.7%)	L – 40 (51.3%)		R – 15 (60%)		L – 10 (40%)	*p* = 0.326
Temporal bone pneumatization volume – L and R (cm^3^)	R – 10.6 (IQR 8.71 – 12,45)	L – 10.65 (IQR 9.40 – 12.1)		R – 14.40 (IQR 12.4 – 17.3)		L – 13.65 (IQR 11.32 – 15.27)	*p* < 0,001*** *p* = 0.002*
Mastoid pneumatization type (*n*; *n*%)	L I – 3 (3.8%) II – 24 (30.8%) III – 30 (38.5%) IV – 21 (26.9%)	R I – 5 (6.4%) II – 17 (21.8%) III – 30 (38.5%) IV – 26 (33.3%)		L I – 2 (8%) II – 1 (4%) III – 12 (48%) IV – 10 (40%)		R I – 1 (4%) II – 3 (12%) III – 11 (44%) IV – 10 (40%)	L – *p* = 0.023** R – *p* = 0.743**
Apex pneumatization type (*n*; *n*%)	L I – 25 (32.1%) II – 42 (53.8%) III – 6 (7.7%) IV – 5 (6.4%)	R I – 23 (29.5%) II – 46 (59%) III – 6 (7.7%) IV – 3 (3.8%)		L I – 2 (8%) II – 10 (40%) III – 9 (36%) IV – 4 (16%)		R I – 1 (4%) II – 11 (44%) III – 9 (36%) IV – 4 (16%)	L - *p* < 0.001** R - *p* < 0.001**

*U-Mann–Whitney Test; **Chi2 Test/Fisher’s exact test; ***T-student test; IQR, interquartile range. Bold text for p statistically significant.

Therefore, a further analysis was conducted taking into consideration the tumor side. The results of this analysis are presented in Table 2. Based on the conducted analysis, it was shown that the volume of temporal bone pneumatization, measured volumetrically, significantly differed between the control and study groups (R: 11.15 [IQR 8.93–12.83] vs. 13.25 [IQR 10.15–15.53]; *p* = 0.040; L: 10.95 [IQR 9.5–12.26] vs. 14.4 [IQR 13.03–15.7]; *p* = 0.012). Furthermore, only apex pneumatization showed significant differences between the control and study groups (R: *p* = 0.033; L: *p* = 0.006). The Dexian Tan classification includes three areas that should be treated collectively, as results in these areas can differ significantly. For instance, a high degree of mastoid pneumatization does not necessarily coincide with a high degree of apex or infralabyrinthine pneumatization. To better represent the overall degree of temporal bone pneumatization according to this classification, we assigned arbitrary cutoff points. Scores up to 6 points were labeled as low pneumatization, scores between 7 and 8 as moderate pneumatization, and scores between 9 and 11 as significant pneumatization. Based on this classification, we conducted an analysis that showed the frequency of pneumatization grades on the right side differed significantly between the study and control groups (*p* = 0.002). However, this association was not observed in patients with left-sided tumors (*p* = 0.088).

**Table 2:** The radiological data considering the operated side.

	Control group (*n* = 78)		CSF Leak group (*n* = 25)		
Side of tumor (*n*%)	R – 38 (48.7%)	L – 40 (51.3%)	R – 15 (60%)	L – 10 (40%)	
Volume of bone pneumatization on side of tumor	11.15 (IQR 8.93-12.83)	10.95 (IQR 9.5-12.26)	13.25 (IQR 10.15-15.53)	14,4 (IQR 13.03-15.7)	R; *p* = 0,040** L; *p* = 0,012*
Volume of tumor	8,54 (IQR 6.46 vs. 14.83)	11.2 (IQR 4.44-16.57)	11.85 (IQR 8.64-15.18)	10.1 (IQR 8.36-16.56)	R; *p* = 0,305* L; *p* = 0,723**
Mastoid pneumatization type (*n*; *n*%)	R I – 3 (7.9%) II – 6 (15.8%) III – 16 (42.1%) IV – 13 (34.2%)	L I – 1 (2.5%) II – 12 (30%) III – 16 (40%) IV – 11 (27.5%)	R I – 1 (6.7%) II – 1 (6.7%) III – 6 (40%) IV – 7 (46.7%)	L I – 0 II – 1 (10%) III – 6 (60%) IV – 3 (30%)	R; *p* = 0,869*** L; *p* = 0,551***
Apex pneumatization type (*n*; *n*%)	R I – 15 (39.5%) II – 19 (50%) III – 3 (7.9%) IV – 1 (2.6%)	L I – 9 (22.5%) II – 23 (57.5%) III – 3 (7.5%) IV – 5 (12.5%)	R I – 1 (5.7%) II – 9 (60%) III – 3 (20%) IV – 2 (13.3%)	L I – 0 II – 3 (30%) III – 5 (50%) IV – 2 (20%)	R; *p* = 0,033*** L; *p* = 0,006***
Infralabyrinthine pneumatization type (*n*; *n*%)	R I – 8 (21.1%) II – 26 (68.4%) III – 4 (10.5%)	L I – 8 (20%) II – 25 (62.5%) III – 7 (17.5%)	R I – 6 (40%) II – 7 (46,7%) III – 2 (13,3%)	L I – 3 (30%) II – 7 (70%) III – 0	R; *p* = 0,357*** L; *p* = 0,391***
Classification total score (*n*; *n*%)	Group I – 9 (23.7%) Group II – 27 (71.1%) Group III – 2 (5.3%)	Group I – 18 (45%) Group II – 15 (37.5%) Group III – 7 (17.5%)	Group I – 1 (6,7%) Group II – 7 (46,7%) Group III – 7 (46.7%)	Group I – 1 (10%) Group II – 7 (70%) Group III – 2 (20%)	R; *p* = 0,002*** L; *p* = 0,088***

*U Mann–Whitney Test; **T-Student Test; ***Chi2/Fisher Exacts Test; IQR, interquartile range. Bold text for p statistically significant.

In the studied group on the left side, the one-way ANOVA indicated that there is a statistically significant difference among subgroups I, II, and III. The average results in group I were lower than those in groups II and III by an average of 3.14 cm^3^ (*p* = 0.001) and 4.86 cm^3^ (*p* < 0.001), respectively. No statistically significant difference was observed between groups II and III (*p* = 0.157).

For the right side, the Kruskal-Wallis test was performed, which revealed a statistically significant difference in medians among the groups (*p* = 0.004). Further pairwise comparison analysis with Bonferroni correction indicated that no difference was observed between subgroups I and II (*p* = 0.610), while a difference was noted between groups I and III (*p* = 0.005) and between groups II and III (*p* = 0.033) (Table 3).

**Table 3:** The results of the average air volume among the groups.

The side on which bone pneumatization was measured (n)	L – 103	R – 103	
pneumatization of the temporal bone depending on group assignment (mean in cm3 + SD)	Group I – 8,99 cm3 (± 2,83) Group II – 12,17 cm3 (± 2,54) Group III – 13,86 cm3 (± 2,41)	Group I 9,29 cm3 (± 4,15) Group II 11,57 cm3 (± 3,36) Group III 14,61 cm3 (± 3,52)	L: *p* < 0,001* R: *p* = 0,004**

*ANOVA with Post-Hoc; **Kruskal Wallis Test; SD, standard deviation. Bold text for p statistically significant.

## Discussion

The main aim of our study was to evaluate the pneumatization of the temporal bone and its potential association with the occurrence of cerebrospinal fluid (CSF) leak. The results of our study showed a statistically significant difference in the volume of temporal bone pneumatization between patients with and without CSF leak. Furthermore, the volumetric values of pneumatization assessed in our study are consistent with the average values reported in the literature (10). This indicates that, in the preoperative phase, the evaluation of temporal bone pneumatization could become a valuable tool—not only in terms of surgical access but also in enhancing the physician’s vigilance during the postoperative period. Importantly, to our knowledge, this is the first study that has morphometrically assessed the relationship between bone pneumatization and the frequency of CSF leak. We divided the classification into three parts, categorizing patients into groups of low, moderate, and significant bone pneumatization. This approach allowed us to assign absolute volume values to each group, enabling physicians to classify patients preoperatively not only through a classification system that could introduce subjective bias since it is based on radiological features but also to relate their own measurements to established literature values. Additionally, our analysis indicated that a higher degree of apex pneumatization was more frequently observed in the group of patients with CSF leak, supporting preliminary findings from the literature (8). However, it remains unclear whether infralabyrinthine pneumatization and mastoid pneumatization play any role in this aspect. To definitively confirm the findings of our study, a prospective study would be necessary to allow for causal inferences.

Currently, two classification systems exist in the literature: our study is based on the classification by Dexian et al., while the second classification by Han et al. evaluates pneumatization in relation to the sigmoid sinus, labyrinth, and carotid canal. Given that previous reports suggest that pneumatization of the petrous apex may influence the incidence of CSF leak, we chose to apply the first classification since it takes apex pneumatization into account. However, our observations indicate that using this classification may be problematic due to the subjective assessment of radiological features of the temporal bone. Moreover, our analysis highlighted that only the petrous apex has an impact on CSF leak. Therefore, relying solely on this classification without incorporating volumetric calculations may prove insufficient as a tool for preoperative evaluation.

While estimating the risk of CSF leak is crucial for the clinical vigilance of the surgeon and for potential intraoperative measures that may protect against postoperative CSF leak, it is important to emphasize that there is no definitive data in the literature indicating which interventions effectively prevent the leaks (11). Some studies have described the use of abdominal fat, wax, and the use of muscle fascia fragments to fill defects as effective measures; however, no prospective studies have been conducted comparing different surgical techniques aimed at preventing CSF leak (12). Based on our research, we did not observe a correlation between the type of surgical reconstruction used and the occurrence of CSF leak. Therefore, in addition to studies estimating the risk of CSF leak, it is also necessary to conduct research that identifies proven and effective prevention methods. Currently, preventive measures primarily rely on the actions taken by the surgeon based on their experience with specific techniques, highlighting the need for further evidence-based guidelines in this area.

We consider the meticulously collected clinical data of patients, along with the assessment of clinical findings, to be an additional asset of our study. This comprehensive approach will enhance the ability to estimate risk more effectively in discussions with patients. Our results are consistent with existing literature regarding transient deficits of the VII cranial nerve (acceptable damage on the House-Brackmann scale grades I-IV was found in 82.5% of our study, compared to 79% reported in the literature), preserving anatomical continuity of the VII nerve (91.26% in our study vs. 82–97% in other series), preoperative hydrocephalus (22.3% vs. 3.7–42% in other series), rates of reoperation, and length of hospitalization (13–15). The slightly higher mortality rate (2.9% vs. 1%) is due to the fact that it refers to a 30-day period, whereas in the literature, it is most often assessed during the perioperative period. However, the data is inconsistent; in some studies, the perioperative period is defined as 7 days, while in others, it extends to 30 days. Additionally, patients who present with significant vascular issues, burdened by multiple comorbidities and requiring urgent surgery, may constitute a larger proportion of our sample. In our group, at the preoperative stage, the group with total hearing loss accounted for 44.7%, patients with serviceable hearing represented 51.5%, and those with good hearing made up 3.8%. Considering only patients who retained hearing, postoperatively, hearing was preserved in 45.6% of them, with all achieving a serviceable level of hearing. According to the literature, the retrosigmoid approach allows hearing preservation in 40–60% of patients over a short observation period. However, it should be noted that hearing preservation is closely related to tumor size, and in our series, the tumors operated on were large, therefore the final percentage of patients with hearing impairment reached 72%. However, this statistics also includes patients who had significant hearing loss prior to surgery. Additionally, it should also be mentioned that the degree of hearing loss changes over time. After 5 years, hearing in the series of patients operated for *VS* using the retrosigmoid approach was reduced to 30–50% (16). These findings complement those in the literature and may serve as a valuable resource for future meta-analyses.

A significant advantage of our study is that the methods used are reproducible and are becoming available at nearly every neurosurgery department worldwide. Volumetric assessment has been successfully applied to numerous classifications and neurosurgical conditions (17–20). Our study indicated that bone pneumatization measured by volumetric methods is feasible, and the data do not differ from those in the literature. Moreover, it does not require specialized programs utilizing algorithms that are impractical in clinical practice. Consequently, we provide a tool that, although requiring prospective validation, can offer important clinical information.

The limitation of our study is its retrospective nature, which means we cannot draw conclusions about causal relationships. However, it indicates that the pneumatization of the temporal bone, particularly the pneumatization of the petrous apex, could be the focus of prospective analyses. Another significant limitation of the study is that it is single-center; however, it is important to note that there are no studies in the field of temporal bone pneumatization using reproducible and widely accessible tools.

## Data Availability

The raw data supporting the conclusions of this article will be made available by the authors, without undue reservation.
